# Pulmonary rehabilitation for pulmonary hypertension in high-altitude areas: a mixed-methods study of medical staff’s perspectives

**DOI:** 10.3389/fpubh.2026.1740477

**Published:** 2026-02-09

**Authors:** Wenrui Li, Yu Zheng, Meng Fan, Yan Sun, Xiuying Li, Zhenfeng Zhao, Xiaozhou Wang, Yaping Zeng, Mingwei Chen

**Affiliations:** 1 Department of Respiratory and Critical Care Medicine, The First Affiliated Hospital of Xi’an Jiaotong University, Xi’an, Shaanxi, China; 2 Department of Respiratory and Critical Care Medicine, Qinghai Special Hospital of Provincial Cardio-Cerebrovascular Diseases, Xining, Qinghai, China; 3 Department of Hypertension, Qinghai Special Hospital of Provincial Cardio-Cerebrovascular Diseases, Xining, Qinghai, China; 4 Department of Coronary Heart Disease, Qinghai Special Hospital of Provincial Cardio-Cerebrovascular Diseases, Xining, Qinghai, China; 5 The Coronary Heart Disease Center, Beijing Anzhen Hospital, Capital Medical University, Beijing, China

**Keywords:** high altitude, medical staff, mixed-methods study, pulmonary hypertension, pulmonary rehabilitation

## Abstract

**Background:**

In high-altitude areas, while pulmonary rehabilitation (PR) is recognized as an effective intervention for enhancing exercise tolerance and reducing breathlessness, delivering these programs to patients living at high altitudes presents unique, environment-specific challenges. This study aimed to systematically investigate the current status, barriers, and optimization pathways for PR in patients with pulmonary hypertension (PH) in high-altitude areas, from the perspective of medical staff.

**Methods:**

A mixed-methods design was employed, comprising a questionnaire survey of 326 medical staff and semi-structured interviews with 16 staff members from three tertiary hospitals in Xining, Qinghai Province. Quantitative data were analyzed using descriptive statistics, and qualitative data were analyzed using thematic analysis.

**Results:**

Quantitative results revealed that only 62.58% of participants reported the implementation of PR in their departments. While 55.39% indicated that they adjusted the content of PR according to the high-altitude environment. Major barriers identified included insufficient awareness among patients and family members (90.80%), lack of high-altitude PR guidelines (88.04%), and shortage of healthcare human resources (87.42%). Optimization suggestions focused on developing high-altitude PR guidelines (86.50%), increasing in rehabilitation resources (82.82%), and improvement of patients’ compliance (78.22%). Qualitative findings identified three core themes: current status of PR and effects, barriers to implementing PR, and optimization suggestions, with 13 subthemes. The quantitative and qualitative findings corroborated each other, indicating insufficient clinical penetration, a lack of standardization, and intertwined multi-level barriers for PR in high-altitude areas. Optimization needs were highly concentrated on guideline development, resource supplementation, technology empowerment, and policy support.

**Conclusion:**

Pulmonary rehabilitation for PH in high-altitude areas exhibits low adoption rates and poor standardization based on medical staff reports, facing a complex barrier system. There is a need to establish an integrated solution centered on high-altitude guidelines, supported by digital technology, and grounded in policy guarantees to facilitate a shift from the current fragmented, experience-based practice toward standardized and systematic services.

## 1 Introduction

Pulmonary hypertension is a cardiopulmonary disease characterized by a progressive increase in pulmonary vascular resistance, potentially leading to right heart failure and death (1). High-altitude areas, due to their elevated levels and low atmospheric oxygen partial pressure, are significant predisposing factors for the onset of PH. Hypoxia can activate pulmonary vascular endothelial cells, promote the release of vasoconstrictive factors, and accelerate pulmonary vascular remodeling, ultimately leading to the development and progression of PH (2–4). Studies have shown that pulmonary arterial pressure in residents of high-altitude areas is significantly higher than in those from lowland areas (5).

Pulmonary rehabilitation, as a core component of the comprehensive management of PH, employs multi-dimensional measures such as breathing training, exercise intervention, and psychological support. It can effectively improve patients’ exercise capacity and quality of life while reducing readmission rates (6, 7). However, the high-altitude hypoxic environment imposes specific requirements on the adaptability and safety of PR protocols. Furthermore, disparities in regional healthcare resources may lead to significant variations in the effectiveness of PR implementation (8, 9). Currently, there is a lack of systematic research on the status of PR for PH in high-altitude regions. In particular, the perceptions, practical barriers, and improvement suggestions of healthcare professionals, who design and execute rehabilitation programs, have not been sufficiently explored. This significantly hinders the optimization and standardization of PR systems for PH in high-altitude areas.

Xining, the capital of Qinghai Province, is located in the northeastern part of the Qinghai-Tibet Plateau with an average altitude of 2,261 meters (10). This study targets medical staff from this region, using a combination of questionnaire surveys and semi-structured interviews, the study systematically collects data on medical staff evaluations of the current PR implementation status, barriers, and optimization pathways. The aim is to identify key issues in the implementation of PR in advanced medical institutions within core high-altitude cities. This will provide an empirical basis for developing standardized PR programs tailored to the characteristics of the high-altitude environment and aligned with regional healthcare resources, thereby ultimately contributing to the improvement of treatment outcomes and quality of life for PH patients across the entire high-altitude region.

## 2 Methods

### 2.1 Study design

This study utilizes a mixed-methods design, integrating both quantitative and qualitative approaches. Ethical approval for the study was obtained (No. QXYYLL-2024-87).

### 2.2 Study participants

This study targeted medical staff from departments managing PH patients in three tertiary hospitals in Xining, which represent the highest level of medical care in the region. The relevant departments primarily included Respiratory and Critical Care Medicine, Cardiovascular Medicine, and other related clinical units involved in PH patient care.

Participants were included based on the following criteria: (1) possession of a valid professional practice certificate; (2) at least 1 year of work experience; (3) experience in treating or caring for patients with PH; and (4) informed consent and willingness to participate in the study. Exclusion criteria included: interns, rotating trainees, and visiting scholars.

### 2.3 Research tools

#### 2.3.1 Quantitative research tools

A self-designed non-scale questionnaire was developed based on the research objectives and a literature review. The initial draft was evaluated by five experts in high-altitude medicine and nursing for content validity, relevance, and clarity. After revisions, a pilot test with 10 medical staff indicated an average completion time of 5.2 min with no reported difficulties, demonstrating good feasibility. The final questionnaire contained 23 items covering four dimensions: participants’ characteristics, current status of PR, aspects of barriers and optimization, and additional opinions (Supplementary Material Questionnaire).

#### 2.3.2 Qualitative research tools

A preliminary interview outline was developed based on the research objectives and consultation with high-altitude clinical medicine and nursing experts. Pre-interviews were conducted with two healthcare professionals. After transcribing the interviews, the research team refined the interview outline regarding relevance, thematic focus, appropriateness of language, and detail of questioning, resulting in a finalized semi-structured interview outline consisting of 9 questions (Supplementary Table S1).

### 2.4 Data collection process

#### 2.4.1 Quantitative data collection

Convenience sampling was used for recruitment, and data collection occurred in April 2025. The questionnaire was administered online using “Wenjuanxing,” a widely used Chinese survey platform. Survey links and QR codes were distributed to potential participants through departmental heads and hospital administrative channels. Informed consent was obtained electronically prior to accessing the questionnaire. The platform incorporated skip logic and mandatory items to ensure data completeness. A small monetary incentive was provided upon submission to encourage participation.

#### 2.4.2 Qualitative data collection

For the qualitative research, purposive sampling was used to extract research subjects from participants who had received a questionnaire survey. Sample size was determined by thematic saturation, defined as no new themes or subthemes emerging from two consecutive interviews. Face-to-face interviews were conducted by a trained researcher (Wenrui Li) in a quiet hospital conference room, each lasting 15–30 min. Another team member (Yu Zheng) observed and took notes. All interviews were audio-recorded with consent, transcribed verbatim within 24 h, and anonymized to ensure confidentiality.

### 2.5 Data analysis methods

#### 2.5.1 Quantitative data analysis

Quantitative data collected via the “Wenjuanxing” platform were exported in Excel format and analyzed using SPSS 26.0. Descriptive statistics were applied, with categorical variables summarized as frequencies and percentages. Figures were generated using Origin 2021.

#### 2.5.2 Qualitative data analysis

The qualitative data were analyzed following Braun and Clarke’s six-step thematic analysis framework (11). To enhance credibility, the primary interviewer and another researcher performed coding and theme development independently. The resulting themes were then cross-verified and finalized through team discussions. NVivo 15 software was utilized to support data management and coding.

#### 2.5.3 Mixed-methods data integration

A complementary analysis of the quantitative and qualitative data was performed. Statistical findings were first used to outline general characteristics, and insights from qualitative themes were then integrated to offer in-depth explanations, enabling the two methods to mutually validate and enrich the findings.

## 3 Results

### 3.1 Quantitative research results

#### 3.1.1 Participant characteristics

A total of 326 valid questionnaires were collected. The detailed characteristics are presented in Table 1.

**Table 1:** General characteristics of participants (*n* = 326).

Item	Frequency (*n*)	Percentage (%)	Cumulative percentage (%)
Sex			
Male	30	9.20	9.20
Female	296	90.80	100.00
Age group			
≤25 years	2	0.61	0.61
26–30 years	38	11.66	12.27
31–40 years	217	66.56	78.83
41–50 years	59	18.10	96.93
51–60 years	10	3.07	100.0
Occupation			
Doctor	69	21.17	21.17
Nurse	257	78.83	100.00
Professional title			
Senior	9	2.76	2.76
Associate senior	31	9.51	12.27
Intermediate	222	68.10	80.37
Junior	64	19.63	100.00
Years of work experience			
1–5 years	28	8.59	8.59
6–10 years	86	26.38	34.97
11–20 years	179	54.91	89.88
≥21 years	33	10.12	100.00

#### 3.1.2 Current status of PR

**Figure 1:**
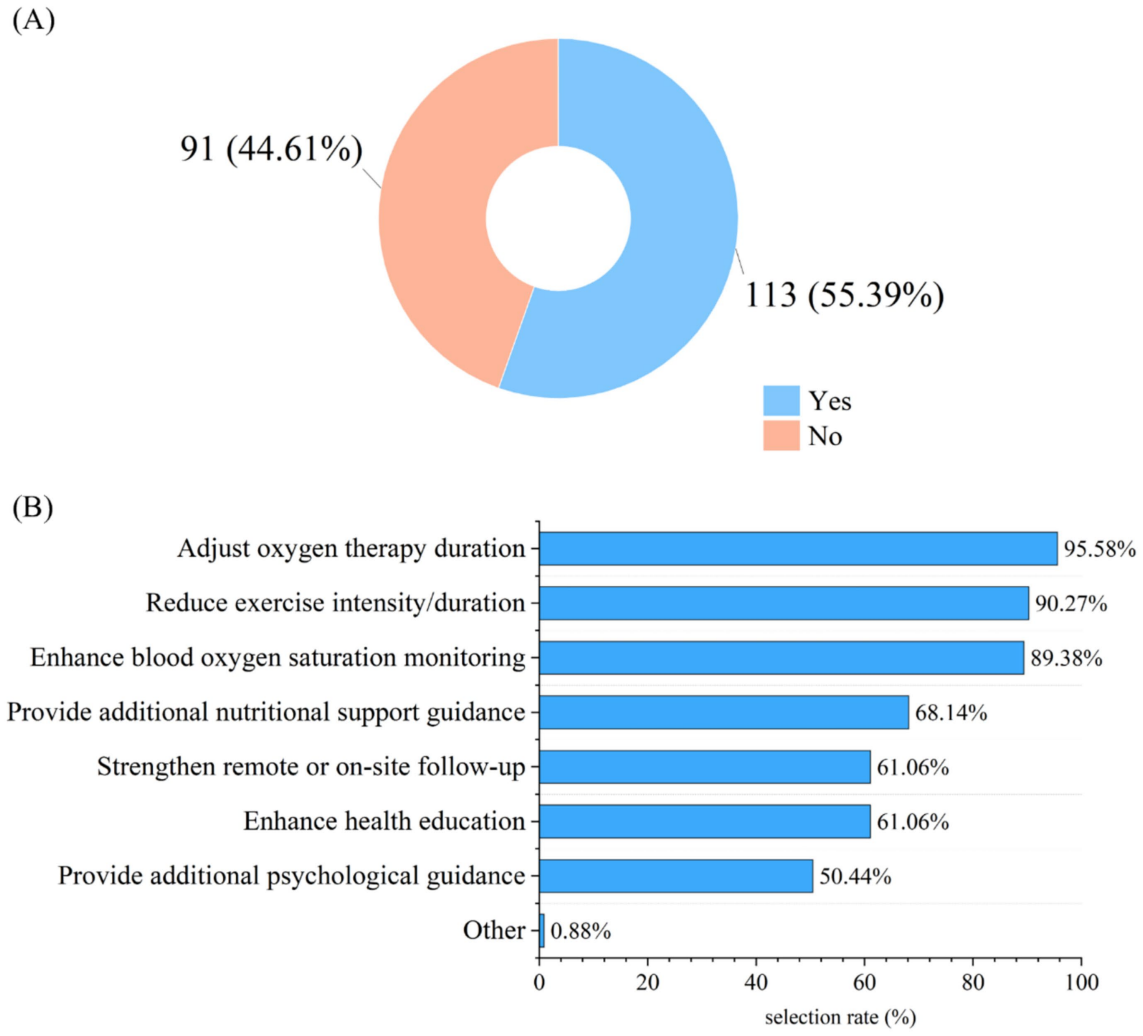
Adjust the content of PR according to the high-altitude environment. **(A)** Assessment of whether PR workflows were adjusted for the special high-altitude environment, as reported by medical staff from implementing departments (*n* = 204). **(B)** Specific aspects of the PR workflows that were modified, based on responses from the 113 medical staff who reported making adjustments (multiple responses allowed).

**Figure 2:**
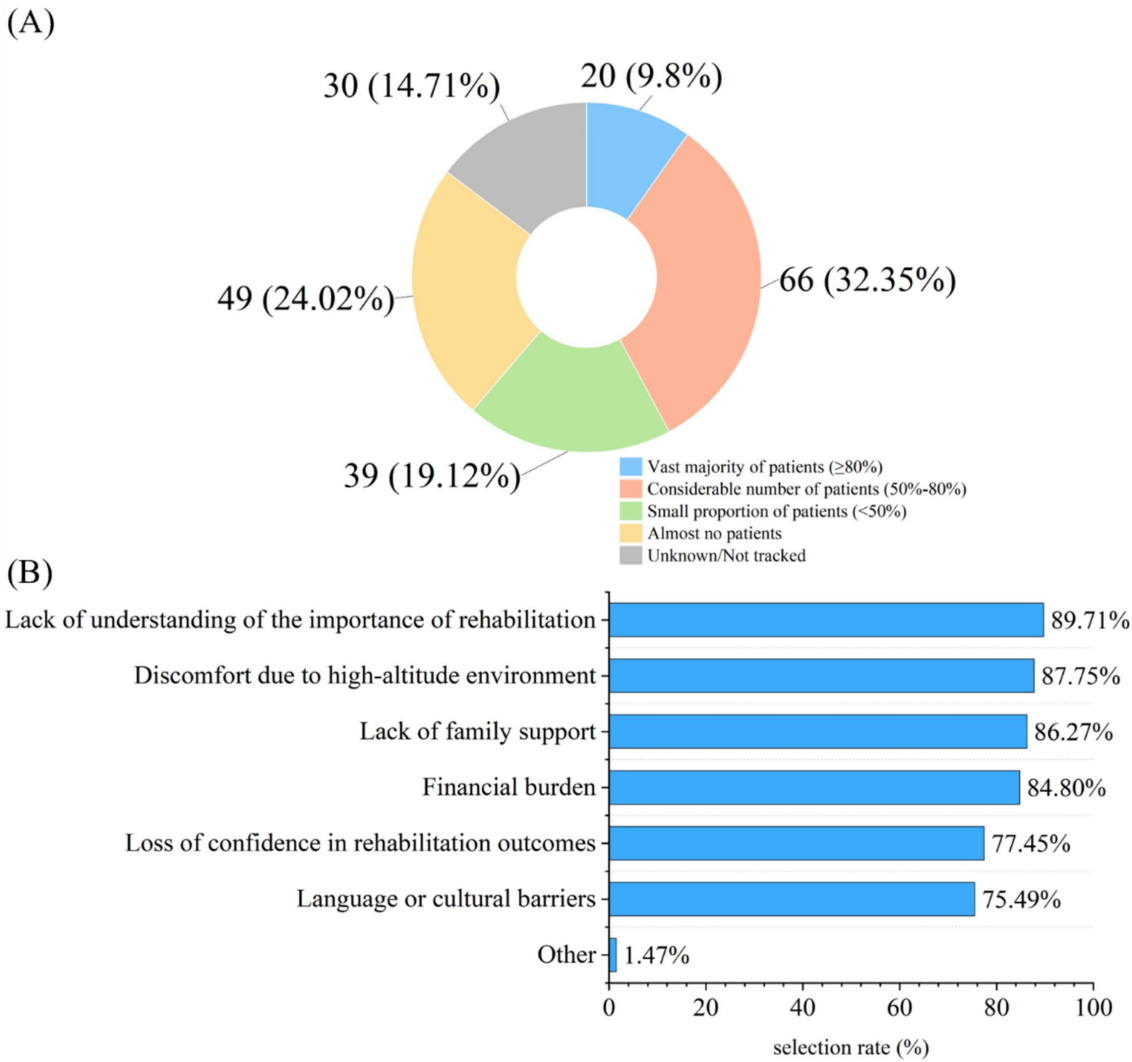
Patient adherence to PR exercises (*n* = 204). **(A)** Proportion of patients who consistently adhered to the rehabilitation exercise regimen, as reported by medical staff. **(B)** Common reasons cited for discontinuation of the rehabilitation exercises, based on reports from medical staff (multiple responses allowed).

Regarding the current status of PR, among 326 surveyed participants, only 204 (62.58%) reported that their departments conducted PR-related work. Further investigation into these 204 participants revealed details about departmental resource allocation and PR effectiveness: 111 (54.41%) indicated that standardized procedures had been developed for these rehabilitation works; in terms of resource allocation, 141 participants (69.12%) stated their departments were equipped with rehabilitation-related equipment (e.g., breathing training equipment, exercise rehabilitation equipment), 129 (63.24%) reported having received special training on PR, and 154 participants (75.49%) noted there were no full-time medical staff dedicated to disease rehabilitation management, while 41 (20.1%) reported 1–2 full-time staff members in their departments. In terms of changes in patients’ symptoms after rehabilitation exercise, 190 participants (93.14%) observed an improvement in patients’ dyspnea, 189 (92.65%) noted an improvement in patients’ fatigue, and 166 (81.37%) reported observing fluctuations in the effectiveness of PR due to altitude differences (Table 2). For altitude adaptation adjustments, 113 participants (55.39%) indicated that PR content had been adjusted to address the high-altitude environment, with the top three adjustment aspects being modification of oxygen therapy duration (95.58%), reduce exercise intensity/duration (90.27%), and enhanced blood oxygen saturation monitoring (89.38%) (Figure 1). Regarding patients’ adherence to PR exercise, 86 participants (42.15%) stated that more than half of the patients could persist in rehabilitation exercise, and the top three reasons for patients’ interruption of rehabilitation exercise were lack of understanding of the importance of rehabilitation (89.71%), discomfort due to high-altitude environment (87.75%), and lack of family support (86.27%) (Figure 2).

**Table 2:** Current status of PR.

Item	Frequency (*n*)	Percentage (%)	Cumulative percentage (%)
Implementation of PR			
Whether the department conducts PR-related work for patients with PH? (*n* = 326)			
Yes	204	62.58	62.58
No	122	37.42	100.00
Whether standardized procedures have been developed for these PR works? (*n* = 204)			
Yes	111	54.41	54.41
No	93	45.59	100.00
Whether the department is equipped with rehabilitation-related equipment? (*n* = 204)			
Yes	141	69.12	69.12
No	63	30.88	100.00
Whether having received special training on PR? (*n* = 204)			
Yes	129	63.24	63.24
No	75	36.76	100.00
Number of full-time medical staff dedicated to disease rehabilitation management in the department (*n* = 204)			
0 staff	154	75.49	75.49
1–2 staff	41	20.10	95.59
≥3 staff	9	4.41	100.00
Observed changes in patients’ symptoms after rehabilitation exercise			
Degree of dyspnea (*n* = 204)			
Significantly improved	96	47.06	47.06
Slightly improved	94	46.08	93.14
No change	8	3.92	97.06
Worsened	6	2.94	100.00
Degree of fatigue (*n* = 204)			
Significantly improved	100	49.02	49.02
Slightly improved	89	43.63	92.65
No change	9	4.41	97.06
Worsened	6	2.94	100.00
Whether observing fluctuations in the effect of PR in patients due to altitude differences? (*n* = 204)			
Yes	166	81.37	81.37
No	38	18.63	100.00

#### 3.1.3 Aspects of barriers and optimization

**Figure 3:**
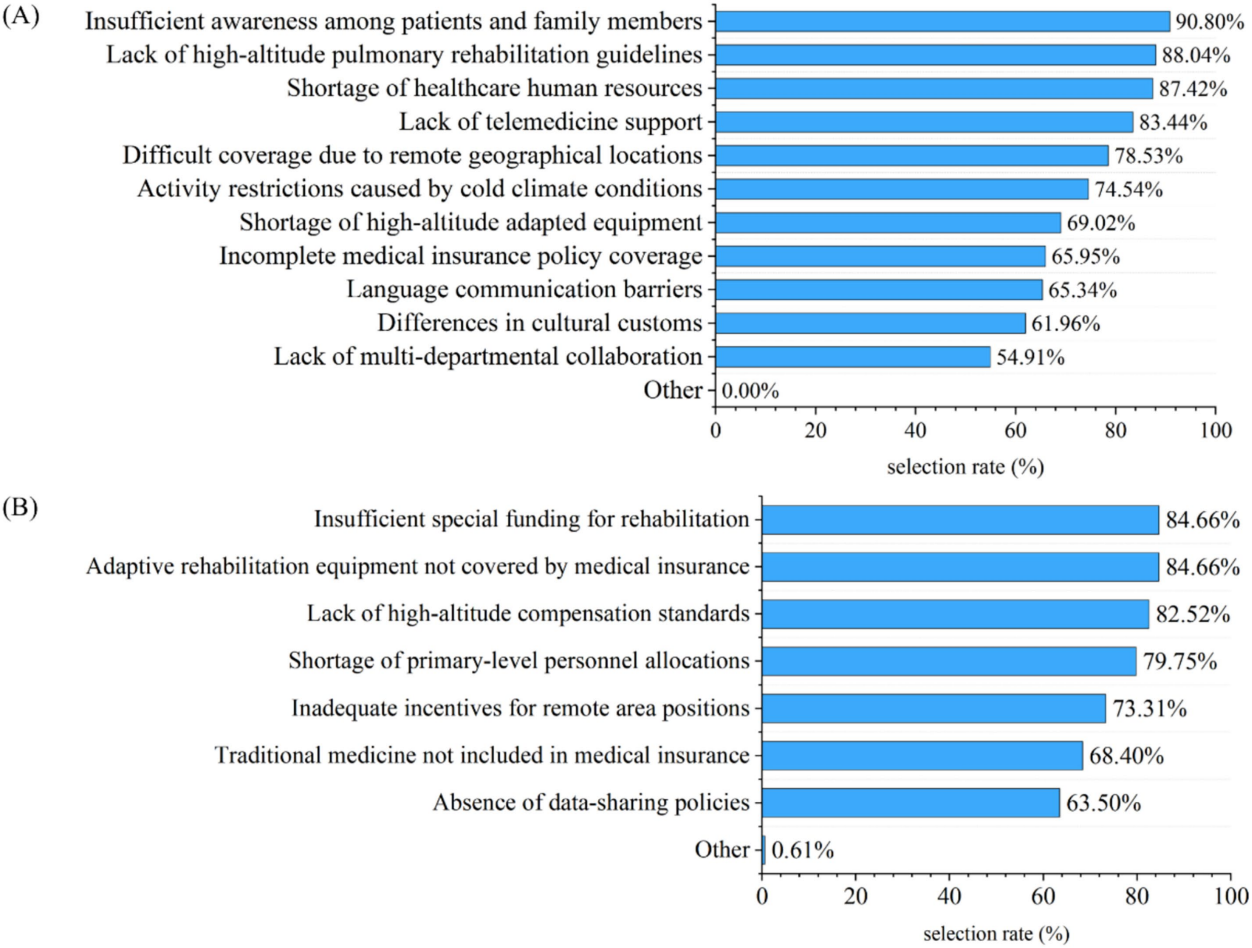
Identified main barrier factors and policy support gaps in PR implementation (multiple responses allowed; *n* = 326). **(A)** Major barrier factors hindering the implementation of PR programs, as reported by medical staff. **(B)** Perceived deficiencies in policy support for PR, as reported by medical staff.

Regarding the main barriers to implementing PR, the primary reasons cited by 326 participants were insufficient awareness among patients and their families (90.80%), lack of high-altitude PR guidelines (88.04%), and shortage of healthcare human resources (87.42%). In terms of policy support deficiencies, the top three issues identified were insufficient special funding for rehabilitation (84.66%), adaptive rehabilitation equipment not covered by medical insurance (84.66%), and lack of high-altitude compensation standards (82.52%) (Figure 3).

**Figure 4:**
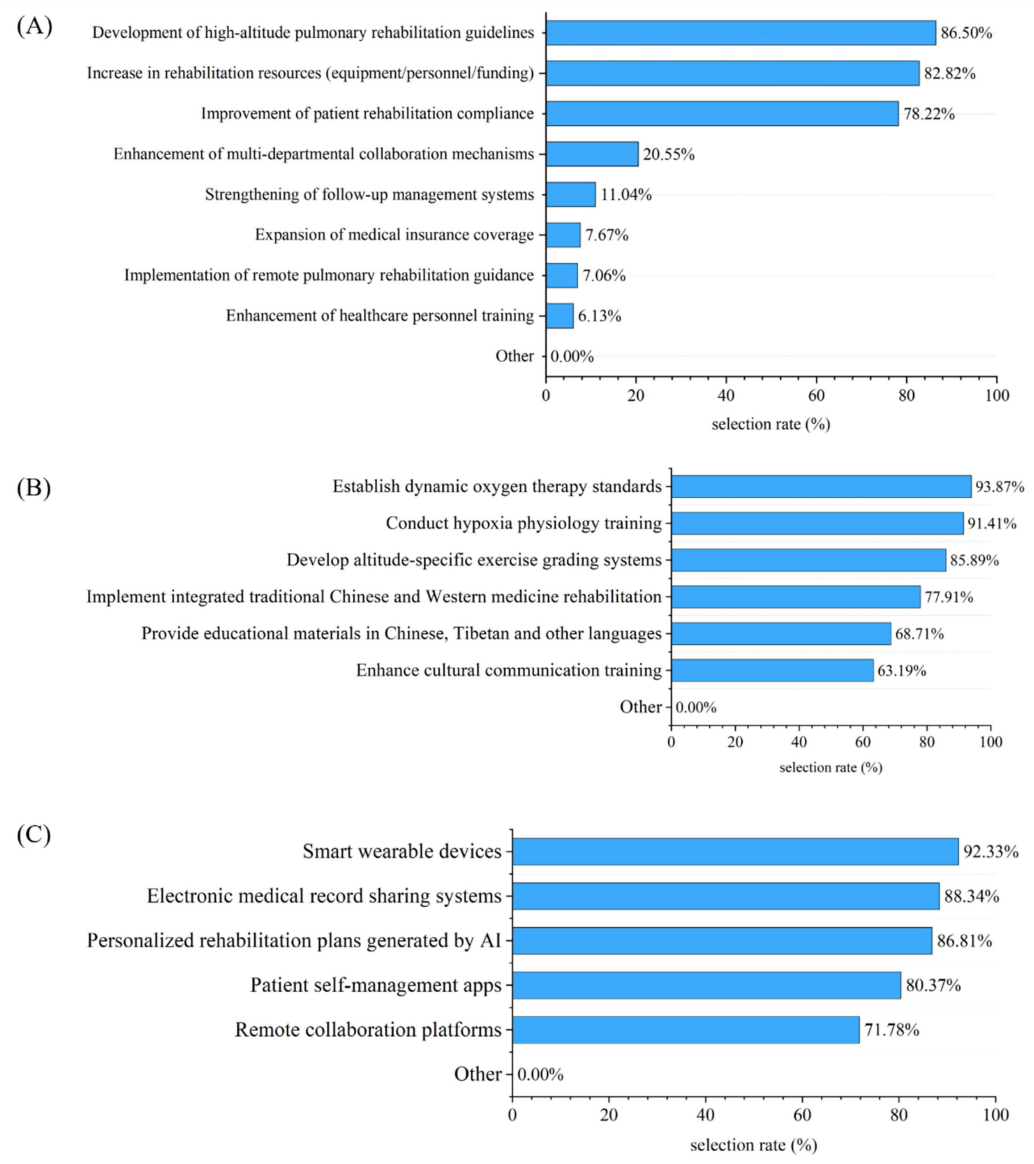
Medical staff recommendations for future improvement of PR programs (multiple responses allowed; *n* = 326). **(A)** The top three priority issues identified by medical staff as requiring urgent solutions. **(B)** Suggested strategies to enhance the adaptation of rehabilitation protocols to the high-altitude environment. **(C)** Technology tools perceived by medical staff as potentially effective in improving patient rehabilitation outcomes.

Optimization suggestions focused on developing high-altitude PR guidelines (86.50%), increasing in rehabilitation resources (82.82%), and improvement of patients’ compliance (78.22%). To improve patient adaptability to high altitudes, the most frequently cited approaches were establishing dynamic oxygen therapy standards (93.87%), conducting hypoxia physiology training (91.41%), and develop altitude-specific exercise grading systems (85.89%). Participants noted that technologies such as smart wearable devices (92.33%), electronic medical record sharing systems (88.34%), and AI-generated personalized rehabilitation plans (86.81%) could effectively enhance patient rehabilitation outcomes (Figure 4).

#### 3.1.4 Additional suggestions or opinions from participants regarding PR services

A total of 27 participants put forth suggestions, which were predominantly manifested in the following seven aspects: increasing professional staffing and enhancing their skill sets; establishing standardized and normative operational frameworks; focusing on the upgrading of hardware and technology; strengthening patient management and health education; enhancing awareness at the societal level; advocating for policy and resource support from higher authorities; and developing targeted intervention measures for specific environments and needs. Detailed descriptions are provided in the Supplementary Table S2.

### 3.2 Qualitative research results

A total of 16 participants were interviewed, including 8 doctors and 8 nurses. Detailed results are shown in Table 3. Through thematic analysis, three core themes and 13 subthemes were extracted.

**Table 3:** Baseline characteristics of interviewed participants.

Number	Sex	Age (years)	Professional title	Educational background	Work experience (years)
Doctor1	Male	47	Associate chief physician	Bachelor’s degree	18
Doctor 2	Male	42	Associate chief physician	Bachelor’s degree	18
Doctor 3	Male	27	Resident physician	Bachelor’s degree	5
Doctor 4	Female	41	Associate chief physician	Bachelor’s degree	17
Doctor 5	Female	38	Attending physician	Bachelor’s degree	13
Doctor 6	Female	37	Attending physician	Bachelor’s degree	14
Doctor 7	Female	34	Attending physician	Bachelor’s degree	12
Doctor 8	Female	30	Resident physician	Master’s degree	5
Nurse 1	Female	51	Associate chief nurse	Bachelor’s degree	30
Nurse 2	Female	45	Associate chief nurse	Bachelor’s degree	20
Nurse 3	Female	34	Supervisor nurse	Bachelor’s degree	15
Nurse 4	Female	30	Supervisor nurse	Bachelor’s degree	9
Nurse 5	Female	38	Supervisor nurse	Bachelor’s degree	14
Nurse 6	Female	35	Supervisor nurse	Bachelor’s degree	10
Nurse 7	Female	43	Supervisor nurse	Bachelor’s degree	17
Nurse 8	Female	29	Supervisor nurse	Bachelor’s degree	8

#### 3.2.1 Theme 1: current status of PR and effects

PR measures and implementation characteristics: The current PR measures for inpatients with high-altitude PH primarily include oxygen therapy, breathing exercises, and physical training. However, the implementation of breathing and exercise training remains fragmented, often limited to “verbal instructions followed by low-intensity, self-guided practice”. This approach lacks standardized, individualized assessment based on objective metrics.

High-altitude adaptation adjustment: interventions aimed at adapting to the high-altitude environment are predominantly empirical. For instance, oxygen therapy duration may be extended without unified target oxygen saturation levels, and exercise rehabilitation is primarily guided by the patient’s subjective tolerance.

Management and follow-up mechanism: post-discharge management is mainly conducted through patient WeChat groups. However, the frequency of home-visit follow-ups is low.

Effect evaluation: medical staff have observed improved pulmonary function in patients with good compliance. Nonetheless, the absence of standardized follow-up procedures and systematic data tracking makes it difficult to comprehensively evaluate the effectiveness of home-based PR. Supporting verbatim quotations for these subthemes are provided in Table 4.

**Table 4:** Current status of PR and effects.

Subtheme	Illustrative
PR measures and implementation characteristics	“Guide the patient to, for example, blow a balloon for about 10–15 min after waking up in the morning, as well as perform pursed-lip breathing, diaphragmatic breathing, and use a respiratory training device. There is no fixed duration, just a standard time frame.” (Doctor 7)
	“Currently, the rehabilitation services provided to patients within the department primarily involve education by doctors and nurses, including activities such as balloon blowing, diaphragmatic breathing, oxygen therapy, and moderate aerobic exercise. The most accessible options at present are balloon blowing and Baduanjin. There is virtually no financial investment required, and these interventions are mainly implemented in the patient’s ward during hospitalization.” (Doctor 8)
	“We teach patients breathing exercises, and our department is currently implementing oxygen-carrying exercises, pursed-lip breathing, and abdominal breathing.” (Nurse 1)
High-altitude adaptation adjustment	“Generally speaking, what we do for this is to increase the duration of oxygen therapy, which is 15 h or more. However, we usually do it for more than 15 h, and for some severe patients, it can even reach about 20 h. And this is the exercise, because they already have a heavy lack of oxygen, and coupled with the low oxygen in a high-altitude area, it may be activity. Compared to low altitude areas, the intensity of these exercises is not as high.” (Doctor 3)
Management and follow-up mechanism	“But now for our nebulized inhalation medication, we have created a WeChat group, which is a follow-up group. We will actively ask him how he is doing and if he has used up the medication? How have you been these past few days?” (Doctor 6)
	“There is a WeChat group like this, and before we schedule, we also distribute some compliance with medication and rehabilitation breathing exercises videos, short videos, to the WeChat group.” (Nurse 1)
	“In terms of follow-up, our responsible nurse will go home to evaluate the effectiveness after discharge… We have now conducted follow-up on 5 patients.” (Nurse 2)
Effect evaluation	“There is a patient with chronic obstructive pulmonary disease and old pulmonary tuberculosis who also has PH. He has a ventilator at home and a home oxygen therapy device. The drugs he inhales and the targeted drugs he uses to lower pulmonary arterial pressure follow the doctor’s advice very well. His quality of life and survival rate have significantly improved.” (Doctor 4)
	“There is currently no corresponding evaluation of the effectiveness of existing rehabilitation treatments, they have only been carried out according to requirements. On the other hand, they are not systematic and comprehensive enough, and there is no dedicated person to complete respiratory rehabilitation.” (Doctor 8)
	“We only gave him these teachings during his hospitalization, but we do not have any evaluation criteria for how he will perform in the later stages.” (Nurse 1)

#### 3.2.2 Theme 2: barriers to implementing PR

Individual and behavioral factors of patients: Insufficient patient understanding of the disease (e.g., fear of “oxygen dependency”), widespread distrust toward doctors, lack of caregiving support for older adult individuals living alone, and language barriers among some ethnic minority patients collectively contribute to poor compliance.

Geographical and environmental factors: The hypoxic high-altitude environment limits patients’ exercise capacity. Remote locations and inconvenient transportation reduce the frequency of medical consultations and increase the difficulty of follow-up.

Economic burden and cost issues: Financial constraints limit patients’ access to quality medical services, rehabilitation equipment, and medications.

Conflict between culture and belief: Cultural beliefs or practices among some ethnic minority patients can impact healthcare seeking and rehabilitation adherence. For instance, some may turn to a Living Buddha for serious illnesses, and some Tibetan patients prefer Tibetan medicine and treatments.

Insufficient medical resources: Inadequate allocation of healthcare staff and shortages of rehabilitation equipment significantly hinder the delivery of PR.

Shortcomings of existing PR programs: The current PR programs lack standardized procedures. Health education content is delivered in a monotonous format, leading to poor information dissemination. Furthermore, there is an absence of clear quantitative indicators and operational guidelines for high-altitude adaptation, as well as a standardized post-discharge follow-up mechanism. Verbatim supporting quotations for these subthemes are provided in Table 5.

**Table 5:** Barriers for implementing PR.

Subtheme	Illustrative
Individual and behavioral factors of patients	“The patient’s compliance is not good, and there are very few who can persist in doing mouth breathing and health exercises……For example, in terms of oxygen therapy, whether it’s a nasal cannula mask or a ventilator, they always feel that they become dependent on it after wearing it for a long time and will not be able to remove it in the future, so they may use it during acute exacerbations. I think it’s more likely to have a higher level of cultural awareness, and I do not think his own understanding of this disease is enough. They adjust the duration of their oxygen therapy according to their own wishes……I think it’s the patients who have decreased their trust in the hospital and doctors. Some patients and their families are not particularly cooperative, so they may have a big discount on compliance, or they may not trust the doctor’s words very much……It is indeed possible that our hospital is in Chinese, and there are few translations of Tibetan language. They do not know how to write, and even with Tibetan language, they do not know it. They only know this language. Therefore, cultural knowledge has an impact on ethnic minorities in remote areas.” (Doctor 1)
	“The old man was alone at home and could not take care of himself, so he had to procrastinate until his children came back to pick him up and take him to the hospital. So the life cycle of these patients is shorter. If there is someone at home to take care of them, their lifespan will also be longer.” (Doctor 2)
	“Inhalation of medication is a long-term chronic treatment. Some patients may not be able to persist and may stop taking medication because they feel better. Many patients give up halfway due to improper use of medication or incorrect inhalation methods, resulting in poor treatment outcomes.” (Doctor 4)
	“I think it’s related to cognition, which is about these Tibetan and Hui pastoral areas. They believe in Buddhism and their own beliefs.” (Nurse 8)
Geographical and environmental factors	“Especially in pastoral areas, there is still a part that is mainly based on grazing, and his place of residence is not fixed, so his compliance may be poor.” (Doctor 5)
	“After the patient returns, the medicine will not be used for a long time, especially because their reason is that it is too far away and we cannot get through.” (Doctor 6)
	“I think this is related to our altitude. Overall, the patient population for pulmonary hypertension is still… the acceptance rate is relatively high among rural and pastoral patients.” (Nurse 2)
Economic burden and cost issues	“There are some patients who do not have their own source of income. Sometimes he cannot hold off until the end because he cannot take it anymore or borrow money (for medical treatment)” (Doctor 6)
	“We (the patients here) buy very few oxygen concentrators, and the conditions at home are indeed very poor. Most of us are poor people here, who are very sick and have to wait for a long time before coming and then getting on a ventilator.”(Nurse 8)
Conflict between culture and belief	“(Patients) will make major decisions, they will go to the living Buddha to decide whether to seek medical treatment or further treatment.” (Doctor 2)
	“Some Tibetan patients find it difficult to receive Western medicine treatment due to their beliefs or customs, and it is also difficult to follow medical advice for corresponding rehabilitation.” (Doctor 8)
	“Regarding their culture, faith, and beliefs. I think this is because we also have more Hui people and people who believe in Buddhism.” (Nurse 8)
Insufficient medical resources	“As for our department, although we have 55 beds, we have a maximum of six non-invasive breathing machines in our general practice, and then add four high flow ventilators.” (Doctor 1)
	“The reimbursement ratio in Qinghai is still relatively low, mainly because the reimbursement ratio for patients is 60%, plus some threshold fees, he may only be able to report 40%.” (Doctor 2)
	“The second one is this rehabilitation exercise. Currently, some of these equipments sometimes cannot keep up.” (Doctor 3)
	“The personnel are indeed quite busy. The respiratory department is quite busy, and in the afternoon, there is a bit of tension. The personnel are really limited because our number of people is really not enough.” (Nurse 4)
Shortcomings of existing PR programs	“There are no specific standards. There is no specific plan given to him, only verbal preaching without a specific implementation process……The publicity is relatively poor. The overall publicity, including our hospital and even the government’s publicity efforts, is not enough, and patients’ understanding of the disease is also insufficient……There is still a lack of clear quantitative indicators and operational norms for adjusting the PR needs and intervention measures of patients at different altitudes……The amount and level of activity that patients engage in must be different. It is necessary to reduce the frequency according to the altitude……There is no complete management plan or standardized follow-up mechanism.” (Doctor 2)
	“There is a process that we drafted ourselves, but this is something we drafted ourselves, and it cannot be said to be completely right or wrong.” (Nurse 7)

#### 3.2.3 Theme 3: optimization suggestions

Optimization of intervention content: It is recommended to develop individualized and differentiated exercise plans based on the patient’s disease severity and tolerance. Long-term home oxygen therapy and early respiratory rehabilitation exercises should be emphasized, alongside strengthened nutritional guidance, psychological support, health education.

Technical tools and methods: Utilize a variety of tools for health education, including brochures, videos, WeChat groups, and instructional videos. However, WeChat group management must be enhanced to ensure information is accurate and practical. Wearable devices like smart wristbands can be widely adopted to provide dynamic feedback for rehabilitation through real-time data monitoring.

Policy and institutional safeguards: Healthcare institutions are advised to establish dedicated respiratory rehabilitation centers equipped with professional rehabilitation devices and to create positions for clinical nutritionists. At the policy level, it is recommended to expand the scope of health insurance coverage and increase reimbursement rates to alleviate the financial burden on patients. Efforts should also promote directing resources toward primary care, implementing disease-specific management for target populations, and ensuring the implementation of relevant clinical guidelines in high-altitude areas. Verbatim supporting quotations for these subthemes are provided in Table 6.

**Table 6:** Optimization suggestions.

Subtheme	Illustrative
Optimization of intervention content	“For those with lung problems in high-altitude areas, we definitely recommend long-term home oxygen therapy.” (Doctor 1)
	“Compared to low altitude areas, I think for oxygen therapy, in our high-altitude region, the oxygen inhalation time may be longer and equipped with this kind of oxygen related equipment.” (Doctor 5)
	“Compared with low altitude areas, it may be necessary to comprehensively consider the economic conditions of patients and provide them with economical and efficient respiratory rehabilitation guidance…It may be necessary to increase psychological intervention. Many patients have anxiety, depression, or sleep problems, which are currently a weakness.” (Doctor 8)
	“Many patients cannot intervene if they cannot tolerate it. Some lie in bed and some stand up, depending on the patient’s condition.” (Nurse 3)
	“Do a good job in evangelism. Go home and stick to oxygen therapy, then have regular outpatient visits, and after that, take medication according to the doctor’s advice. The inhaled medication is basically not stopped. In Tibetan areas, people do not pay attention to their diet because they already eat a lot of beef and mutton, so they need to pay attention to their diet.” (Nurse 6)
Technical tools and methods	“WeChat groups will invite patients to join, and corresponding professional technicians will answer your questions every day.” (Doctor 1)
	“In terms of technical tools, we can now use portable devices such as oxygen saturation monitoring and heart rate monitoring, like wristbands, to dynamically observe patients’ rehabilitation.” (Doctor 3)
	“For example, recording a video… You can send it to his phone and watch it for a long time when he goes back. Follow this video to practice together.” (Doctor 4)
	“Actually, we also add some WeChat accounts for patients and their families now. They can always tell us anything, just like playing the role of a family doctor. If there is a dedicated platform and personnel to manage this aspect, it may be better……With the help of remote or Internet, or the small brochures, we can follow up outside the hospital.” (Doctor 5)
	“But this is because if this group becomes bigger and bigger, he will send it to you all day and night, and it’s not good if there is no one to manage it.” (Nurse 1)
	“Join the WeChat group or make a phone call. If they are older, they play with their phones less, and then family members can tell them.” (Nurse 6)
	“The video may be the simplest. The most commonly used Tiktok or Kwai video, which he may brush while watching, will definitely pay attention to.” (Nurse 7)
Policy and institutional safeguards	“Whether it’s oxygen concentrators or ventilators, if the country can keep up with the support, it will definitely be a good thing for lung rehabilitation.” (Doctor 1)
	“Also, there is a rehabilitation specialist specifically for patients with pulmonary hypertension who can provide them with training……The policy of prevention as the main focus, as well as the Healthy China policy, mainly targets many chronic diseases. If we start managing and gradually promoting them from the grassroots level, it may have a certain effect.” (Doctor 3)
	“We can also provide some equipment, such as specialized respiratory training devices. It depends on the patient’s wishes, and then, how convenient they are, which one they are willing to choose, and then provide them with the corresponding equipment.” (Doctor 4)
	“I think our respiratory patients need to have a nutritionist.” (Doctor 5)
	“Guidelines and expert consensus related to high-altitude areas need to be implemented, and public welfare publicity needs to be strengthened.” (Doctor 8)
	“I hope we can have a rehabilitation department, so that all of us older adult patients with pulmonary heart disease can go to the department for more comprehensive and professional rehabilitation training.” (Nurse 8)

### 3.3 Integrated interpretation of results

The integrated quantitative and qualitative findings collectively delineate the current landscape, barriers, and potential optimization pathways for PR in high-altitude regions.

Regarding the current state of PR practice, the quantitative data (62.58% of participants reported PR implementation in their departments) and qualitative descriptions (characterizing the measures as being in a “fragmented stage”) jointly indicate insufficient penetration and systematization of PR within clinical practice. Both methodological approaches revealed significant gaps in departmental resource allocation.

Concerning high-altitude adaptation adjustments, the quantitative result (55.39% of participants reported making adjustments to PR content) and qualitative finding (that these are “primarily based on empirical adjustments”) mutually corroborate each other. This convergence demonstrates that while adaptation practices are common, they lack standardization.

In terms of implementation barriers, the top-ranked obstacles identified in the quantitative survey—such as insufficient patient awareness, lack of specific guidelines, and inadequate resources—were richly elaborated and contextualized through specific examples and detailed explanations provided in the qualitative interviews.

Pertaining to optimization pathways, a high degree of consensus emerged from both research methodologies. The core needs expressed by quantitative participants—for developing high-altitude-specific guidelines and increasing resource allocation—directly correspond to the recommendations derived from qualitative analysis, which emphasized addressing the lack of standardized procedures and optimizing the configuration of human resources and equipment.

## 4 Discussion

This study systematically elucidates the current status, barriers, and optimization pathways of PR practices for PH in high-altitude areas from the perspective of medical staff. By integrating quantitative and qualitative data, the research not only quantifies existing gaps in practice but also provides an in-depth analysis of the multiple challenges faced by frontline clinicians in the unique high-altitude environment, along with their adaptive strategies. The findings offer indispensable evidence for constructing a feasible PR framework.

### 4.1 Systemic deficiencies: practical bottlenecks and the need for standardization

This study found that despite the widely recognized clinical value of PR (12, 13), its adoption rate and systematic implementation are severely inadequate in high-altitude areas. The fact preliminarily indicates poor clinical penetration of PR in these regions. More critically, qualitative interviews revealed that these practices often remain fragmented, typically limited to “verbal education plus low-intensity, self-guided exercise”. This low level of systematization is directly linked to inadequate resource allocation: lacked full-time rehabilitation management staff, and qualitative interviews frequently cited a severe shortage of rehabilitation equipment. This finding aligns with the “resource-practice” disconnect commonly faced in the early stages of PR promotion across many regions (14, 15), a problem whose consequences are amplified in the unique high-altitude environment (16, 17). When confronting the distinctive high-altitude environment, our study uncovered a prevalent pattern of “experiential adaptation.” The participants reported modifying PR content, such as extending oxygen therapy or reducing exercise intensity. However, these adjustments lacked objective, quantitative criteria, such as dynamic oxygen saturation thresholds. This indicates that while clinicians are aware of the environmental specificities, they lack evidence-based guidelines to translate this experiential knowledge into standardized protocols. The absence of such standardization may not only compromise rehabilitation outcomes but also pose potential risks due to inappropriate adjustments (18). Therefore, developing evidence-based clinical pathways for high-altitude PR and defining its core parameters (e.g., the relationship between exercise intensity and oxygen saturation levels) is a crucial step toward advancing PR practice in these regions from an experiential to a scientific foundation.

### 4.2 Multi-level implementation barriers: from individual to systemic

This study clearly outlines a multi-level barrier system spanning from micro to macro dimensions. At the patient level, both quantitative and qualitative data identify insufficient awareness among patients and their families as a primary obstacle. Qualitative interviews provide deeper insights: patient misconceptions about the disease (e.g., “oxygen dependency”), lack of social support for older adult individuals living alone, and language and cultural barriers for ethnic minority patients collectively form a complex psychosocial network leading to poor compliance. This suggests that future interventions must move beyond simple knowledge dissemination and require culturally adapted, population-specific health education strategies. At the healthcare system level, a chain of deficiencies in “human resources-standardized tools-follow-up support” constitutes the major bottleneck. Staff shortages are an objective reality, but the lack of standardized operating procedures, training systems, and follow-up mechanisms prevents limited human resources from achieving their maximum potential. This aligns with findings from multiple studies implementing complex interventions in resource-limited settings (19–21). At the environmental and geographical level, the high-altitude hypoxic environment itself is perceived as a fundamental barrier. It not only directly limits patient function (22, 23), but also exacerbates rehabilitation interruptions by triggering “high-altitude environment discomfort”. Geographical remoteness and transportation difficulties further increase the challenge of follow-up and management, making it difficult for healthcare staff to effectively monitor discharged patients (24–26). At the macro-policy level, economic burden is a core barrier hindering the implementation of PR. This study indicates that insufficient dedicated funding, the non-inclusion of rehabilitation equipment in health insurance, and high long-term costs directly restrict treatment accessibility and continuity. This “financial toxicity” forces many patients to discontinue rehabilitation due to cost concerns (27, 28). Consequently, any optimization of techniques or protocols will have limited impact without supporting economic and policy measures (29, 30).

### 4.3 Optimization pathways: technology integration, personalization, and policy support

Despite severe challenges, this study identifies clear and actionable directions for future interventions. First, the urgent need for the development of high-altitude PR guidelines, as expressed by medical staff, should be a top priority. These guidelines must include quantifiable assessment and intervention standards specifically tailored to the high-altitude environment. Second, technology-enabled solutions show significant potential. Healthcare staff highly recognize the value of smart wearable devices, electronic medical record systems, and AI-generated personalized plans. These tools can facilitate remote monitoring, overcoming geographical barriers, and support the creation and dynamic adjustment of individualized regimens through objective data, thereby partially alleviating human resource shortages (31, 32). However, qualitative interviews also highlighted a caveat: poorly managed digital tools (e.g., WeChat groups) could lead to information chaos. Therefore, the introduction of technology must be accompanied by well-defined operational management procedures. Finally, all optimization recommendations point to the necessity of systemic reform. Suggestions range from establishing dedicated PR centers and adding professionals like nutritionists, to formulating specific high-altitude PR guidelines, promoting health insurance policy reforms, and ensuring the decentralization of resources. Collectively, these form a multi-dimensional optimization framework spanning clinical practice to health policy. Notably, developing “precision rehabilitation” management strategies targeted at specific ethnic, geographical, and age groups is essential to address the demographic diversity of high-altitude regions.

The advantage of this study lies in its use of a mixed-method research design, which systematically explores the current status of PR for PH in high-altitude areas. Key gaps in practice were quantified through quantitative research, and the complex causes behind these phenomena were deeply explained through qualitative interviews, achieving triangulation between data and enhancing the depth and credibility of the findings. Additionally, the study focuses on the key implementation group of healthcare service providers, offering indispensable insights for developing practical and feasible intervention strategies in the future. There are several limitations to this research. First, participants were recruited exclusively from three tertiary hospitals in Xining, and convenience sampling via departmental heads and hospital administrative channels precluded precise calculation of the recruitment denominator or response rate. These factors limit the generalizability of findings to grassroots institutions in plateau regions or other geographical settings. Second, nurses comprised 78.83% of the quantitative sample. Although nurses are central to PR delivery, this imbalance may bias results toward nursing perspectives on practical implementation and patient education, while underrepresenting physician views on decision-making or diagnostics. Given the exploratory design and small physician subsample, no formal subgroup analyses (e.g., by profession or experience) were conducted, as they would have been underpowered. Thirdly, the questionnaire options may not have covered all practical situations, which could lead to a certain degree of bias in the research perspective and potential response bias. Furthermore, all data reflect medical staff perceptions via self-reports rather than direct patient outcomes, clinical records, or objective measures, which may introduce recall or social desirability bias. Finally, despite achieving thematic saturation based on consecutive interviews yielding no new themes, the qualitative sample size (*n* = 16) and diversity remain relatively limited.

## 5 Conclusion

This study indicates a significant supply-demand gap in PR practice for PH in high-altitude areas. Although clinical needs are clear, both popularity and standardization are low. High-altitude adaptation adjustment mainly relies on experience rather than objective evidence. The obstacle is a complex ecosystem composed of patient cognition/culture, system resources, policy guarantees, and high-altitude environments. Therefore, the fundamental path to promote development lies in building a “guideline-technology-policy” trinity solution. This approach provides a technical core through the development of high-altitude guidelines, uses intelligent wearable devices for remote management, and leverages medical insurance coverage and resource investment as system guarantees to achieve a fundamental transformation from empirical practice to a standardized and systematic mode.

## Data Availability

The raw data supporting the conclusions of this article will be made available by the authors, without undue reservation.
