# Supplementary material for: Pulmonary rehabilitation for pulmonary hypertension in high-altitude areas: a mixed-methods study of medical staff’s perspectives
Source: Front Public Health. 2026 Feb 9;14:1740477. doi: 10.3389/fpubh.2026.1740477 (PMC12926409; doi:10.3389/fpubh.2026.1740477)
Supplement: Supplementary file 1 [file Table_1.docx]

**Supplementary Material**

Questionnaire on the Pulmonary Rehabilitation Status of Patients with Pulmonary Hypertension in High-Altitude Areas (*For* *Medical Staff*)

Dear medical staff,

Hello! We are currently conducting a survey on the pulmonary rehabilitation status for patients with pulmonary hypertension in high-altitude areas. This study aims to understand the pulmonary rehabilitation current practices, barriers, and optimization pathways in the unique high-altitude environment, so as to provide a basis for developing more scientific and comprehensive pulmonary rehabilitation plans.

Your participation is highly important! This survey is anonymous and will not disclose any of your personal information. All data will be used solely for academic research. The questionnaire will take approximately 5-8 minutes to complete. Your valuable feedback will directly contribute to improving pulmonary rehabilitation services for pulmonary hypertension patients in high-altitude areas, benefiting more patients. We sincerely appreciate your support and cooperation!

Friendly reminder: Complete the survey to receive a cash reward. Click below “I agree” to start filling it out ↓

1. What is your biological sex?

male

female

1. Which age group do you belong to?

≤25 years

26–30 years

31–40 years

41–50 years

51–60 years

≥61 years

1. What is your occupation?

Doctor

Nurse

1. What is your professional title?

Senior

Associate Senior

Intermediate

Junior

1. How long have you been working?

1–5 years

6–10 years

11–20 years

≥ 21 years

1. Whether the department conducts pulmonary rehabilitation-related work for patients with pulmonary hypertension? (Select "yes" to continue to the next question, or select "no" to skip to Question 18.)

Yes

No

1. Whether standardized procedures have been developed for these pulmonary rehabilitation works?

Yes

No

1. Whether the department is equipped with rehabilitation-related equipment?

Yes

No

1. Whether having received special training on pulmonary rehabilitation?

Yes

No

1. Number of full-time staff dedicated to disease rehabilitation management in your department?

0 staff

1-2 staff

3 or more staff

1. How severe was the patient's dyspnea after the pulmonary rehabilitation exercise?

Significantly improved

Slightly improved

No change

Worsened

1. How severe was the patient's fatigue after the pulmonary rehabilitation exercise?

Significantly improved

Slightly improved

No change

Worsened

1. Whether observing fluctuations in the effect of PR in patients due to altitude differences?

Yes

No

1. Whether to adjust the content of pulmonary rehabilitation work according to the high-altitude environment? (Select "yes" to continue to the next question, or select "no" to skip to Question 16.)

Yes

No

1. What specific adjustments does the content include? (Multiple choice)

Enhance blood oxygen saturation monitoring

Strengthen remote or on-site follow-up

Adjust oxygen therapy duration

Enhance health education

Reduce exercise intensity/duration

Provide additional nutritional support guidance

Provide additional psychological guidance

Other (please specify)

1. What proportion of patients persist with pulmonary rehabilitation exercises?

Vast majority of patients (≥80%)

Considerable number of patients (50%-80%)

Small proportion of patients (<50%)

Almost no patients

Unknown/Not tracked

1. What are the common reasons for patients to discontinue rehabilitation exercises? (Multiple choice)

Lack of understanding of the importance of rehabilitation

Financial burden

Lack of family support

Discomfort due to high-altitude environment

Loss of confidence in rehabilitation outcomes

Language or cultural barriers

Other (please specify)

1. What do you consider to be the main barrier factors in carrying out rehabilitation work? (Multiple choice)

Lack of high-altitude pulmonary rehabilitation guidelines

Shortage of healthcare human resources

Insufficient awareness among patients and family members

Incomplete medical insurance policy coverage

Shortage of high-altitude adapted equipment

Lack of telemedicine support

Difficult coverage due to remote geographical locations

Activity restrictions caused by cold climate conditions

Language communication barriers

Lack of multi-departmental collaboration

Differences in cultural customs

Other (please specify)

1. What are the identified shortcomings in current policy support? (Multiple choice)

Adaptive rehabilitation equipment not covered by medical insurance

Lack of high-altitude compensation standards

Insufficient special funding for rehabilitation

Shortage of primary-level personnel allocations

Inadequate incentives for remote area positions

Absence of data-sharing policies

Traditional medicine not included in medical insurance

Other (please specify)

1. What do you consider to be the three most pressing issues that need to be prioritized in pulmonary rehabilitation in high-altitude areas? (Multiple choice, Select 3 options.)

Development of high-altitude pulmonary rehabilitation guidelines

Expansion of medical insurance coverage

Implementation of remote pulmonary rehabilitation guidance

Enhancement of multi-departmental collaboration mechanisms

Increase in rehabilitation resources (equipment/personnel/funding)

Improvement of patient rehabilitation compliance

Strengthening of follow-up management systems

Enhancement of healthcare personnel training

Other (please specify)

1. How do you think we can enhance the high-altitude adaptability of pulmonary rehabilitation programs? (Multiple choice)

Establish dynamic oxygen therapy standards

Provide educational materials in Chinese, Tibetan and other languages

Develop altitude-specific exercise grading systems

Enhance cultural communication training

Conduct hypoxia physiology training

Implement integrated traditional Chinese and Western medicine rehabilitation

Other (please specify)

1. Which technological tools do you believe can effectively enhance patient rehabilitation outcomes? (Multiple choice)

Electronic medical record sharing systems

Smart wearable devices

Patient self-management apps

Personalized rehabilitation plans generated by AI

Remote collaboration platforms

Other (please specify)

1. Do you have any other opinions or suggestions regarding pulmonary rehabilitation services? (Optional)

Table S1 Interview guide.

| Number | Content |
| --- | --- |
| 1 | What pulmonary rehabilitation services does your institution provide for patients with pulmonary hypertension? What are the standard operating procedures for these rehabilitation measures? Where is the implementation site? |
| 2 | Have standard pulmonary rehabilitation measures been adjusted for high-altitude hypoxic environments? How was it adjusted? |
| 3 | How effective are the existing pulmonary rehabilitation measures? Please provide examples to illustrate. |
| 4 | What do you think are the reasons that affect patients' compliance with pulmonary rehabilitation? Please provide examples to illustrate. |
| 5 | Are there any patients whose pulmonary rehabilitation outcomes are affected by cultural beliefs (such as religious beliefs) or traditional therapies (such as Tibetan medicine)? Please provide examples to illustrate. |
| 6 | What do you think are the limitations and shortcomings of current pulmonary rehabilitation methods? |
| 7 | What other aspects do you think should be included in the pulmonary rehabilitation plan for patients with pulmonary hypertension in high-altitude areas? What aspects need to be adjusted compared to low altitude areas? |
| 8 | What technical tools do you think can improve pulmonary rehabilitation outcomes? |
| 9 | What measures or policies do you think can promote the implementation of the pulmonary rehabilitation plan? |

Table S2 Additional suggestions or opinions from participants regarding PR services.

| Respondent ID | Participant suggestions |
| --- | --- |
| 22 | Improve patient compliance, enhance the professional skills of healthcare workers, and establish corresponding work models and systems. |
| 31 | Increase training for professionals and add necessary equipment. |
| 36 | Strengthen the integration of nutrition into rehabilitation training in high-altitude environments. |
| 43 | Enhance training and optimize staffing. |
| 65 | Professional rehabilitation therapists should conduct pulmonary rehabilitation for patients in high-altitude areas. |
| 71 | Increase the number of pulmonary rehabilitation professionals. |
| 90 | Develop high-altitude pulmonary rehabilitation guidelines. |
| 103 | Establish dedicated pulmonary rehabilitation teams. |
| 121 | Introduce AI technology support. |
| 137 | Promote health education to help patients recognize the importance of pulmonary rehabilitation. Hospitals should establish respiratory therapy departments, train respiratory therapists, provide cardiopulmonary rehabilitation training, offer more professional guidance to patients, familiarize them with various instruments, and leverage technological advancements to improve pulmonary rehabilitation. |
| 154 | Increase follow-up through multiple methods. |
| 173 | Strengthen public health education. |
| 176 | Foster multi-department and multi-institution collaboration. |
| 203 | Enhance standardized training. |
| 204 | Increase policy support. |
| 235 | Strengthen publicity efforts. |
| 244 | Conduct extensive publicity and provide financial and information support. |
| 259 | Develop professional guidelines, train specialized talent, enhance rehabilitation education, increase reimbursement rates, and improve communication. |
| 268 | Encourage healthcare workers to participate in more training and learning opportunities. |
| 276 | Improve knowledge dissemination and enhance patient compliance. |
| 284 | Strengthen health education and raise public awareness of pulmonary rehabilitation. |
| 310 | Enhance personnel training efforts. |
| 312 | Improve infrastructure development. |
| 316 | Hospitals should upgrade relevant facilities and conditions. |
| 320 | Raise awareness and strengthen training. |
| 322 | Accelerate the establishment of cardiopulmonary rehabilitation departments in hospitals and increase staffing. |
| 325 | Further improve patient compliance with medical advice. |
